# Sociodemographic correlates of 25-hydroxyvitamin D test utilization in Calgary, Alberta

**DOI:** 10.1186/1472-6963-14-339

**Published:** 2014-08-09

**Authors:** Lawrence de Koning, Dan Henne, Paul Woods, Brenda R Hemmelgarn, Christopher Naugler

**Affiliations:** 1 Department of Pathology and Laboratory Medicine, University of Calgary, Calgary, AB, Canada; 2 Calgary Laboratory Services, Calgary, AB, Canada; 3 Department of Family Medicine, University of Calgary, Calgary, AB, Canada; 4 Department of Medicine, University of Calgary, Calgary, AB, Canada; 5 C414, Diagnostic and Scientific Centre, 9, 3535 Research Road NW, T2L 2 K8 Calgary, AB, Canada

**Keywords:** Vitamin D, Laboratory utilization, Canada census data

## Abstract

**Background:**

Increasing laboratory test utilization is a major challenge facing clinical laboratories.

However, in most instances we lack population level information on the patient groups to which increased testing is directed. Much recent work has been published on the sociodemographic correlates of 25-hydroxyvitamin D deficiency. An unanswered question, however, is whether testing is preferentially directed towards individuals with a higher likelihood of deficiency. In this paper we examine this question by combining laboratory information system data on testing rates with Census Canada data.

**Methods:**

We examined 1,436 census dissemination areas within the city of Calgary, Alberta, Canada. For each census dissemination area we determined age and sex-specific 25-hydroxyvitamin D testing rates over a one year period. We then compared these testing rates with the following sociodemographic variables obtained from Census Canada: first nations status, education level, household income, visible minority status, and recent immigrant status.

**Results:**

Overall, 6.9% of males in the city of Calgary were tested during the study period. Females were 1.7 times more likely to be tested than males. Testing rate increased with increasing age, with 16.8% of individuals 66 years and over tested during the one-year study period.

Individuals having at least some university education were less likely to be tested (RR = 0.60;

p < 0.0001). Interestingly, although visible minorities were over twice as likely to be tested as compared to non-visual minorities (RR = 2.25; p < 0.0001), recent immigrants, a group known to exhibit low 25 hydroxyvitamin D levels, were significantly less likely to be tested than non-recent immigrants (RR = 0.72; p = 0.0174). While median household income was modestly associated with increased testing (RR = 1.02; p < 0.0001), First Nations status and non-English speaking were not significant predictors of 25-hydroxyvitamin D testing.

**Conclusions:**

Testing for 25-hydroxyvitamin D is in part directed toward populations at higher risk of deficiency (visible minorities) and at higher risk of osteoporosis (older females), but a particularly high risk group (recent immigrants) is being tested at a lower rate than other patient groups.

## Background

Increasing test utilization is one of the major challenges facing laboratories in the 21st century [1], and is compounded by the fact that between 10% and 50% of these laboratory tests may be unnecessary [2]. It is not surprising, therefore, that utilization management has become a major area of interest to laboratories throughout the western world [3-5]. Despite this, there remain many gaps in our understanding of which population groups are being tested at increased or decreased rates and if testing is directed towards groups most likely to benefit. Recent studies of the sociodemographic variables associated with 25-hydroxyvitamin D deficiency provide the opportunity to examine this question by comparing testing rates among sociodemographic groups with and without higher rates of deficiency. For example, we have previously demonstrated a large variation in mean 25-hydroxyvitamin D levels among census dissemination areas in Calgary Alberta [6], with variation in 25-hydroxyvitamin D levels associated with age, immigrant status, education, and income.

In this paper we examine recent testing rates among Census Dissemination Areas in Calgary, Alberta to determine which sociodemographic factors are associated with greater testing rates. It is hoped that a comparison of these relative testing rates with known correlates of low 25-hydroxyvitamin D level will shed light on the appropriateness of current testing effort.

## Methods

### Data sources

We first sought to determine the number of patients of each age and sex group in each census dissemination area who had at least one 25-hydroxyvitamin D test during the study period. 25-hydroxyvitamin D test data were obtained from the Laboratory Information System of Calgary Laboratory Services, the sole provider of laboratory testing for Calgary, Alberta and surrounding areas. 25-hydroxyvitamin D results were included for the period of 11 May 2010 and 10 May 2011. This date range was chosen because it encompasses the 12 month period leading up to the 2011 Canadian Census. For each test result we recorded subject age, sex, date of testing and Provincial Health Card Number. Provincial Health Card Number was then used as a linking variable to obtain postal codes from an Alberta Health Services database. The postal codes were converted to their corresponding census dissemination area and geographic coordinates using a Statistics Canada Postal Code Conversion File at the Spatial and Numeric Services Department of the University of Calgary Library. Census dissemination areas are the lowest geographic level in the Canadian Census and consist of geographical groupings of 300–800 people. Following conversion to dissemination areas, the provincial health card number was removed from the dataset, thereby permanently de-identifying the data. Finally, the following sociodemographic variables were extracted from the 2006 Canada Census (the last year for which this data was available) for each census dissemination area: first nations status, education level (percent of individuals with at least some university education), median household income, visible minority status, and recent immigrant status (immigration within the past 5 years) [7].

### 25-hydroxyvitamin D tests

All 25-hydroxyvitamin D tests used in this study were performed as part of routine patient care. Clinical samples were analyzed in a single laboratory at Calgary Laboratory Services using the LIASON 25-hydroxyvitamin D Total assay (Diasorin, Ltd.). External quality assurance on these assays is performed through subscription to the Vitamin D External Quality Assessment Scheme (DEQAS). The lower limit of detection of this assay was 10 nmol/L. Where more than one test existed for the same subject, one test was chosen at random for that subject and the others were removed from the dataset.

### Statistical analysis

Statistical analysis was conducted in SAS v. 9.3. A negative binomial regression model was used to examine the associations with testing rate, and to infer relationships with neighbourhood level sociodeomgraphic variables. Because of the hierarchical nature of the data we used generalized estimating equations in the model. For the categorical variable sex, “male” was arbitrarily chosen as the reference variable and for age group “age = 66+” was arbitrarily chosen as the reference. For the variables “non-English speaking”, “recent immigrant”, “aboriginal status”, and “visible minority status” the relative risk refers to the presence of this variable as opposed to his absence. For the single continuous variable considered, “median household income”, the relative risk refers to the change in the rate of testing for every $10,000 CDN increase in median household income. The reported relative risks refer to the independent contribution of each variable with the other categorical variables held constant at their reference value and the continuous variable held constant at its mean value. Analyses were considered statistically significant at an alpha of 0.05.

### Ethics statement

This study was approved by the University of Calgary Conjoint Research Ethics Board (ID: E23919).

## Results

A total of 93, 967 individuals (60, 435 females and 33, 532 males) had a least one 25-hydroxyvitamin D test performed in the 12 month study period and were included in the study. The mean (SD) 25-hydroxyvitamin D level was 70.72 nmol/L (34.7 nmol/L). The mean age (SD) was 48.4 years (18.4 years). Tested individuals resided in 1,436 different CDAs within the city of Calgary.

Overall, 6.9% of males in the city of Calgary were tested during the study period. The regression model showed that females were more likely to be tested than males (RR = 1.72). The most commonly tested age group was 66 years and older with 16.8% of individuals tested. Younger individuals were significantly less likely to be tested (Table 1). Among sociodemographic variables, individuals with higher education levels (at least some University education) were less likely to be tested (RR = 0.60), as were recent immigrants to Canada (RR = 0.70). Visible minorities were more likely to be tested (RR = 2.24), as were individuals from higher income households (RR = 1.02). First Nations status and non-English speaking were not significant predictors of 25-hydroxyvitamin D test utilization. Table 2 compares the known correlates of lower 25-hydroxyvitamin D level with the actual testing rates observed in this study. For age, sex, recent immigrant status and income level increased testing was directed toward individuals likely to have higher levels of 25-hydroxyvitamin D, while for education level and visible minority status increased testing was directed toward individuals likely to have lower levels of 25-hydroxyvitamin D.

**Table 1:** Poisson multiple regression analysis of the association of sociodemographic variables with 25-hydroxyvitamin D testing rate

**Variable**	**Relative risk**	**RR 95% confidence limits**		**Chi-square**	**P-value**
**Non-English speaking**	1.0266	0.5089	2.0708	0.01	0.9415
**Recent immigrant (less than 5 years)**	0.6959	0.5162	0.9382	5.66	0.0174
**Aboriginal status**	1.2928	0.8429	1.9828	1.39	0.2392
**At least some university education**	0.6039	0.5028	0.7252	29.15	<.0001
**Visible minority status**	2.2488	1.8993	2.6627	88.39	<.0001
**Median household income**	1.0229	1.0169	1.0289	57.13	<.0001
**Sex = F**	1.7169	1.6889	1.7454	4137.6	<.0001
**Sex = M**	1.0000	reference	reference	reference	reference
**Age = 0-20**	0.1759	0.1682	0.1839	5869.1	<.0001
**Age = 21-45**	0.4459	0.4311	0.4612	2202.1	<.0001
**Age = 46-65**	0.7678	0.748	0.7882	391.91	<.0001
**Age = 66+**	1.0000	reference	reference	reference	reference

Generalized estimating equations were used because of the hierarchical nature of the data.

**Table 2:** Comparison of sociodemographic correlates of lower 25-hydroxyvitamin D levels with testing rates observed in this study

**Variable associated with lower vitamin D level**	**References**	**Was this variable associated with increased testing?**
**Recent immigrant**	[6,8]	No
**Visible minority**	[9-15]	Yes
**First nations status**	[16]	No
**Non English speakers**	N/A	No
**Lower education**	[6,17]	Yes
**Lower income**	[6]	No
**Male sex**	[6,9]	No
**Young adults**	[6,9]	No

## Discussion

In previous work on the same population, we demonstrated that lower 25-hydroxyvitamin D levels were associated with lower income, lower education and immigrant status [6]. The fact that the measurement of 25-hydroxyvitamin D is medically unnecessary in the vast majority of patients [18], led us to suspect that the increase in 25-hydroxyvitamin D test requests in recent years may have been driven by increased personal health advocacy (increased patient requests) and therefore may have led to excess testing of patients at lower risk.

Our findings are somewhat contradictory. On the one hand the higher risk groups of visible minorities and lower education level individuals were tested at a higher rate, but the remainder of the variables showed associations of increased testing with variables associated with higher 25-hydroxyvitamin D levels. For example, young adults have the lowest mean 25-hydroxyvitamin D levels [6,9] but were less than half as likely to be tested than individuals over the age of 65. It is tempting to infer that this may have stemmed from increased testing in patient groups suspected of having a higher risk of osteoporosis. However as females and older individuals are more likely to consult with a primary care physician, [19] the increased testing rates observed in these populations may stem simply from the increased opportunity to receive a test requisition. Alternatively, because the finding of low levels of 25-hydroxyvitamin D in younger individuals has only recently been described and has not been widely disseminated, this may represent a lack of awareness on the part of ordering practitioners.

Our data suggested increased testing among individuals with visible minority status, a known marker of low 25-hydroxyvitamin D [9-15]. However it is concerning that although refugees to the City of Calgary show very low 25-hydroxyvitamin D levels [8], recent immigrants in general are less likely to be tested. One possible explanation for this is that it represents a racial disparity in health care delivery [20-23]. Alternatively it may simply reflect a lack of access to family physicians for new arrivals to the City of Calgary. Congruent with this interpretation, we did not observe an effect of language (at least when comparing English versus non-English speaking), suggesting that being a recent arrival rather a language barrier may be the more important factor associated with lower testing rates. Indeed, reduced access to health care for recent immigrants has been previously described in the Canadian setting [24,25]. Recent immigrants tend to use ad hoc walk-in services soon after arrival in Canada and later transition to regular sources of care [26]. The evaluation of this situation in Calgary is complicated by the fact that there are marked differences among Calgary neighbourhoods in terms of the density of immigrant populations [6] and neighbourhood-level access differences to primary care for immigrants has been shown in another Canadian setting [27], as has neighbourhood level differences in pap-testing rates for immigrant women [28]. It may very well be that decreased health care access for new immigrants leads to increasing use of ad hoc medical services where discretionary laboratory tests and preventative health care interventions are less likely to occur.

The observation of a minor positive association between testing and income is particularly interesting as individuals of higher socioeconomic status are more likely to take dietary supplements [29,30] and so we may have expected this group to also be more likely to request 25-hydroxyvitamin D testing from their physicians. In contrast, although education level is also associated with socioeconomic status, we observed a negative association of testing with higher education.

This research must be interpreted in light of its limitations. First of all, it must be remembered that like all studies utilizing ecological data, inferences regarding group level variables may not necessarily reflect individual level variables. Secondly, it is likely that rates of 25-hydroxyvitamin D ordering were influenced by variation in access to primary care physicians among the various demographic groups. This information was not available to us and therefore must remain a limitation of this study. Third, we do not have information on whether 25-hydroxyvitamin D test requests were based on physician opinion or were patient requested. Research performed in the Canadian province of Ontario demonstrated that patient factors were relatively unimportant in determining discretionary laboratory testing, while physician factors such as specialty, practice volume and previous testing patterns were strongly associated with test ordering [31]. Whether a similar situation exists in Alberta is a question for future research.

Further work could expand this methodology to include other analytes with suspected over-utilization or unexplained variance in ordering patterns. A comparison of populations at risk with the actual associations with increased testing could help to identify laboratory tests with a mismatch between testing pattern and clinical utility.

## Conclusions

Utilizing an ecological approach to matched laboratory and Census Canada data, we found that 25-hydroxyvitamin D test ordering was increased in some groups at higher risk for 25-hydroxyvitamin D deficiency but decreased in other groups at higher risk.

## Competing interests

The authors declare no relevant competing interests.

## Authors’ contributions

CN conceived of the study and collected the data. All authors contributed to the study design. LD and CN performed the analyses and drafted the manuscript. All authors contributed to revisions, read, and approved the final manuscript.

## Pre-publication history

The pre-publication history for this paper can be accessed here:

http://www.biomedcentral.com/1472-6963/14/339/prepub
